# **β-**cell dynamics in type 2 diabetes and in dietary and exercise interventions

**DOI:** 10.1093/jmcb/mjac046

**Published:** 2022-08-05

**Authors:** Chengan Lv, Yuchen Sun, Zhe Yu Zhang, Zeyad Aboelela, Xinyuan Qiu, Zhuo-Xian Meng

**Affiliations:** Department of Pathology and Pathophysiology and Metabolic Research Center of the Second Affiliated Hospital, Zhejiang University School of Medicine, Hangzhou 310003, China; Key Laboratory of Disease Proteomics of Zhejiang Province, Zhejiang University School of Medicine, Hangzhou 310058, China; Department of Pathology and Pathophysiology and Metabolic Research Center of the Second Affiliated Hospital, Zhejiang University School of Medicine, Hangzhou 310003, China; Key Laboratory of Disease Proteomics of Zhejiang Province, Zhejiang University School of Medicine, Hangzhou 310058, China; Zhejiang University–University of Edinburgh Institute (ZJE), Zhejiang University, Haining 314400, China; Department of Pathology and Pathophysiology and Metabolic Research Center of the Second Affiliated Hospital, Zhejiang University School of Medicine, Hangzhou 310003, China; Key Laboratory of Disease Proteomics of Zhejiang Province, Zhejiang University School of Medicine, Hangzhou 310058, China; Department of Pathology and Pathophysiology and Metabolic Research Center of the Second Affiliated Hospital, Zhejiang University School of Medicine, Hangzhou 310003, China; Key Laboratory of Disease Proteomics of Zhejiang Province, Zhejiang University School of Medicine, Hangzhou 310058, China; Bachelors of Surgery, Bachelors of Medicine (MBBS), Zhejiang University School of Medicine, Hangzhou 310003, China; Department of Biology and Chemistry, College of Liberal Art and Sciences, National University of Defense Technology, Changsha 410073, China; Department of Pathology and Pathophysiology and Metabolic Research Center of the Second Affiliated Hospital, Zhejiang University School of Medicine, Hangzhou 310003, China; Key Laboratory of Disease Proteomics of Zhejiang Province, Zhejiang University School of Medicine, Hangzhou 310058, China; Department of Geriatrics, Affiliated Hangzhou First People's Hospital, Zhejiang University School of Medicine, Hangzhou 310006, China

**Keywords:** pancreatic β-cell, type 2 diabetes, dietary intervention, exercise intervention

## Abstract

Pancreatic β-cell dysfunction and insulin resistance are two of the major causes of type 2 diabetes (T2D). Recent clinical and experimental studies have suggested that the functional capacity of β-cells, particularly in the first phase of insulin secretion, is a primary contributor to the progression of T2D and its associated complications. Pancreatic β-cells undergo dynamic compensation and decompensation processes during the development of T2D, in which metabolic stresses such as endoplasmic reticulum stress, oxidative stress, and inflammatory signals are key regulators of β-cell dynamics. Dietary and exercise interventions have been shown to be effective approaches for the treatment of obesity and T2D, especially in the early stages. Whilst the targeted tissues and underlying mechanisms of dietary and exercise interventions remain somewhat vague, accumulating evidence has implicated the improvement of β-cell functional capacity. In this review, we summarize recent advances in the understanding of the dynamic adaptations of β-cell function in T2D progression and clarify the effects and mechanisms of dietary and exercise interventions on β-cell dysfunction in T2D. This review provides molecular insights into the therapeutic effects of dietary and exercise interventions on T2D, and more importantly, it paves the way for future research on the related underlying mechanisms for developing precision prevention and treatment of T2D.

## Introduction

Over recent years, the incidence of type 2 diabetes (T2D) has undergone a rapid global rise (Zhou et al., 2016) and is widely acknowledged as one of the world's most serious public health problems. T2D is characterized by insulin resistance, hyperglycemia, and the eventual lack of insulin secretion from β-cells (Defronzo, 2009). The long-term adverse effects of hyperglycemia including micro- and macro-vascular complications (DeFronzo et al., 2015) can lead to heart disease, stroke, and diabetic retinopathy.

Following the pathogenesis of T2D, pancreatic β-cell dysfunction features as a key aspect at the early stage, whereas an overall loss of β-cell mass is well established as a primary feature of later stages (Eizirik et al., 2020; Weir et al., 2020). As one of the major players in glycemic control, β-cells secrete insulin to induce glucose uptake by the peripheral tissues, therefore controlling glycemic levels (Rorsman and Braun, 2013). In the course of T2D pathogenesis, β-cells undergo a process from functional compensation to decompensation. In prediabetic conditions, moderate hyperglycemia and peripheral insulin resistance do not immediately result in the progression of T2D due to the β-cell compensatory response (Prentki and Nolan, 2006), where increases in β-cell mass and insulin secretion are competent in meeting the elevated demand for insulin (Jetton et al., 2005). However, damage to β-cells begins initiated via chronic escalating metabolic stresses such as oxidative stress, endoplasmic reticulum (ER) stress, and/or inflammation responses (Burgos-Morón et al., 2019). As these factors continue, β-cells and their ability to compensate for such stresses are increasingly compromised so that T2D begins to develop in an increasingly unhindered manner.

T2D can be prevented, managed, and even cured by lifestyle interventions (Knowler et al., 2002; Lean et al., 2018), among which dietary and exercise interventions have long attracted the attention of researchers. The success of dietary- and exercise-based clinical trials has inspired researchers to dig into the underlying mechanisms of these interventions. Subsequent animal- and human-based studies have provided significant progress in our understanding. However, such an understanding is far from complete.

In this review, we mainly focus on the recent advances in revealing the mechanism driving the dynamic alternations of pancreatic β-cells. First, we discuss the key factors and pathways contributing to the failure or decompensation of pancreatic β-cells. We then turn to summarize the effects and mechanisms of dietary intervention and physical exercise on mitigating β-cell dysfunction in T2D.

## Physiological regulation of β-cell insulin secretion

Appropriate insulin synthesis and secretion by β-cells are their critical functions, which represent one of the most important mechanisms mediating glycemic control. Under normal conditions, insulin is translated to pre-proinsulin and transported into the ER lumen. This is followed by the removal of the signal peptide, folding, and the formation of disulfide bonds. Proinsulin is then transferred to the Golgi apparatus, where it is split into C-peptides and insulin, both of which are then stored in secretory granules until their release is triggered by high blood glucose levels (Fu et al., 2014).

The most important regulator of insulin production by pancreatic β-cells is glucose. The glucose-stimulated insulin secretion (GSIS) pathway, the earliest discovered and best-understood insulin secretion process, contributes mostly to insulin secretion. In the GSIS pathway, elevated glycemia activates the membrane glucose transporter, GLUT1 in humans (or GLUT2 in rodents) (Fu et al., 2014). Glucokinase (GK) then phosphorylates intracellular glucose into glucose 6-phosphate, which is a critical rate-limiting step for glucose metabolism in β-cells. Glycolysis produces pyruvate, NADH, and a small quantity of adenosine triphosphate (ATP) from glucose in the cytoplasm. The pyruvate and NADH produced are then transported into the mitochondria and are processed via the tricarboxylic acid (TCA) cycle and electron transport chain (ETC), during which a larger amount of ATP is produced. The ATP/ADP transport complex then transports these ATPs into the cytoplasm. The ATP/ADP ratio in the cytoplasm rises as a result of glycolysis, mitochondrial respiration, and the ETC deactivates ATP-sensitive potassium (K^ATP^) channels and depolarizes the cell membrane. These processes give rise to the activation of voltage-dependent Ca^2+^ channels, which trigger Ca^2+^ influx (Rorsman and Ashcroft, 2018). The increase in intracellular Ca^2+^ concentration facilitates the priming and fusing of insulin granules to the plasma membrane, and insulin exocytosis follows (Rorsman and Ashcroft, 2018).

Direct insulin-secretion-augmentative effects exerted by amino acids and fatty acids can be at least partially attributed to the classical K^ATP^ channel pathway as described above. Amino acids absorbed by β-cells can also enter this pathway through a catabolic process, forming the intermediate products of the TCA cycle. It is important to note that only specific combinations of amino acids can achieve this function (Sener and Malaisse, 1980). For instance, leucine changes the metabolic pathway of glutamate to produce α-ketoglutaric acid, which can then enter the TCA cycle (Sener and Malaisse, 1980). Similarly, upon specific interaction with cell membrane receptors, fatty acids in the blood enter β-cells and become activated into acyl-CoA at the mitochondrial outer membrane, followed by delivery into mitochondria via a carnitine shuttle system. In the mitochondrial matrix, acyl-CoA is oxidized to yield the resultant product, which is used to create ATP via the TCA cycle and ETC.

**Figure 1:**
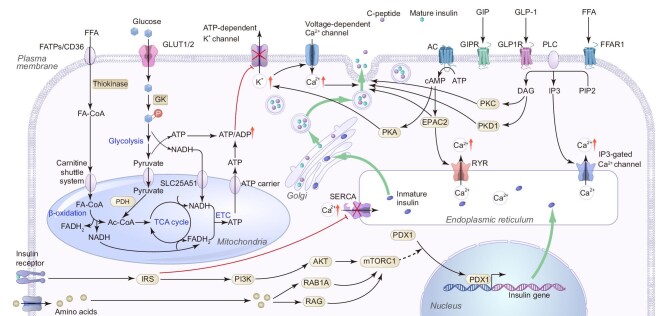
Schematic diagram of the regulation of insulin secretion in β-cells. Glycolysis- and mitochondrial oxidative phosphorylation-mediated elevation of ATP level induces first-phase insulin secretion through inactivation of the K^ATP^ channel, membrane depolarization, opening of the voltage-dependent Ca^2+^ channel, influx of Ca^2+^ from the extracellular space, and the final triggering of the docking of insulin granules on the plasma membrane and insulin secretion. The first phase of insulin secretion is followed by the second phase of insulin secretion that is controlled by the amplifying pathways, in which the ER regulation of intercellular Ca^2+^ homeostasis plays an important role. Incretins, such as GIP, GLP-1, and FFA, can induce insulin secretion through modulating the above glucose-stimulated insulin secretion pathway, such as sensitizing the K^ATP^ channel, adjusting the intracellular Ca^2+^ concentration, and promoting the fusion of insulin granules and the plasma membrane.

In addition, amino acids, fatty acids, and gastrointestinal peptides alter insulin synthesis and secretion through what are known as ‘amplifying pathways’, which are independent of the traditional K^ATP^ channel mechanisms (Figure 1). These pathways, which were discovered later and are still only partially understood, are seen to considerably improve cell secretion function, particularly in the second phase of insulin secretion. Mechanistic target of rapamycin complex 1 (mTORC1) is essential for cellular amino acid sensing (Jewell et al., 2013). Amino acids activate mTORC1 in β-cells via Rag and Rab1A, which promotes the entry of the transcription factor pancreatic and duodenal homeobox 1 (PDX1) into the nucleus and therefore boosts the expression of the insulin gene (Zhang et al., 2021). GPR40, also known as FFAR1, is a fatty acid receptor strongly expressed in the pancreas (Itoh et al., 2003). The receptor activates phospholipase C, which, upon its attachment to external fatty acid molecules, catalyzes the hydrolysis of PIP2 to IP3 or to DAG. IP3 then binds to and opens the IP3-gated Ca^2+^ channel on the ER to release Ca^2+^ storage in the ER, increase the concentration of Ca^2+^ in the cytoplasm, and promote the fusion of insulin granules and the cell membrane (Ferdaoussi et al., 2012). Concurrent to this, DAG activates both PKC and PKD1 (Ferdaoussi et al., 2012), which in turn initiate downstream signaling pathways to result in enhanced insulin granule exocytosis. GIP and GLP-1, secreted by intestinal K cells and L cells, respectively, are two essential incretin hormones that also act on β-cells (Drucker, 2001). They bind to specific receptors on β-cells and mediate adenylate cyclase to convert ATP into cyclic adenosine monophosphate (cAMP). There is evidence that cAMP activates both PKA and Epac2 in islet β-cells. On one hand, PKA sensitizes K^ATP^ channels to augment insulin secretion. On the other hand, activated Epac2 induces the opening of ryanodine receptor (RyR), a Ca^2+^ channel in the ER, in a Ca^2+^-influx-dependent manner (Holz, 2004). Similar to the effect of IP3, Ca^2+^ released from the ER enhances the calcium response of β-cells. There is also evidence that activated Epac2 binds to Rim2 (Kashima et al., 2001), Piccolo, and Rab3 (Ozaki et al., 2000) to induce the release of insulin.

Secreted insulin is found to affect β-cells in an autocrine manner, and the insulin signaling pathway has been shown to be critical for β-cell functionality (Leibiger et al., 2008). Insulin binds to receptors on the membrane of β-cells that have intrinsic tyrosine kinase properties, such as the insulin receptor (IR) (A type and B type) and the insulin-like growth factor-1 receptor (IGF-1R); afterwards, the receptor can be auto-phosphorylated. The activated receptor then phosphorylates adaptor proteins, which include insulin receptor substrates 1–4 (IRS1–IRS4) and Shc in mouse β-cells but only IRS1 and IRS2 in human β-cells. Adaptor proteins activate multiple downstream signaling proteins, including extracellular signal-regulated kinase 1/2 (ERK1/2), phosphatidylinositol 3-kinase (PI3K), AKT, mTORC1, p70S6 kinase, and PLCγ. The majority of the previous studies believe that insulin can promote its own synthesis and secretion. Nevertheless, the effect of insulin on its own synthesis and secretion did not reach a consistent conclusion (Leibiger et al., 2008). This inconsistency is suggested to be caused by the duration and concentration of insulin treatment and the characteristics of the islet microenvironment, such as the glycemic levels, reflecting the dual role of insulin under the conditions of normal insulin signal and hyperinsulinemia (Rachdaoui, 2020). Under physiological conditions, insulin promotes its transcription via the PI3K/p70S6 kinase and CaM kinase pathways (Leibiger et al., 1998). Besides, insulin is reported to promote its own secretion by increasing the concentration of intracellular Ca^2+^, which is probably mediated via various pathways, including the instantaneous IRS1/PI3K-dependent pathway (Aspinwall et al., 2000), and the later suppression of sarco/endoplasmic reticulum Ca^2+^-ATPase (SERCA) by IRS1 (Xu et al., 2000). Furthermore, since insulin can activate mTORC1 via the PI3K–AKT pathway, mTORC1–PDX1-mediated insulin expression is also a possible insulin autocrine regulation pathway (Leibiger et al., 2008; Zhang et al., 2021).

## Mechanisms of β-cell dysfunction in T2D

Results of genome-wide association studies on determining potential T2D-related genetic loci revealed a far larger number of associated genetic variants related to impaired β-cell function compared to those related to insulin resistance (Voight et al., 2010). The role of β-cells in the pathogenesis of T2D has therefore been highlighted as a key area of research over the last decade. Representative observations include (i) a strong association of T allele of the transcription factor 7-like 2 (TCF7L2) Rs7903146 polymorphism with β-cell dysfunction in T2D (Lyssenko et al., 2007), (ii) many observations on the decline in functional β-cell mass as a hallmark trait of T2D, and (iii) significantly lower β-cell mass, smaller islets, and fewer islet equivalents in the pancreas from T2D postmortem studies. Such phenomena have also been readily observable in mouse T2D models, although different strains of mice have shown varying degrees of cell damage. Overall, these results confirm that a progressive decrease in the number and function of β-cells is strongly correlated with the disease progression of T2D.

### The dynamic alterations of β-cell function in T2D disease progression

**Figure 2:**
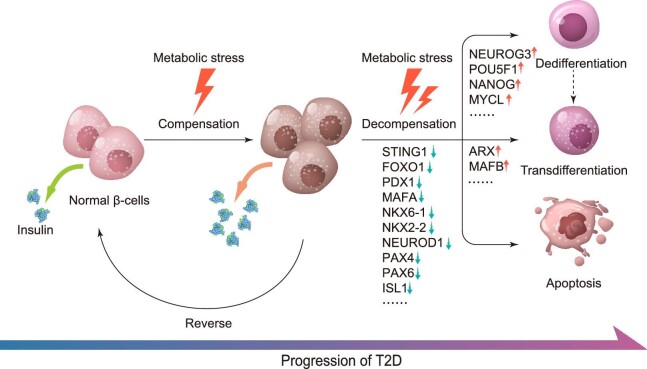
Schematic diagram of β-cell functional alternations under metabolic stress. Metabolic stress first stimulates β-cells to initiate compensatory responses, including hyperplasia, hypertrophy, upregulation of insulin synthesis and secretion, etc. These compensatory responses do not cause irreversible damage to β-cells and can be reversed upon reduction of metabolic stress. However, under prolonged and intense metabolic stress, a cluster of genes critical for β-cell function, such as FOXO1, PDX1, MAFA, and NKX6-1, are downregulated, resulting in functional impairment and decompensation of β-cells. During this process, β-cells also undergo dedifferentiation, transdifferentiation, and eventually apoptosis, leading to the gradual onset of T2D.

The highly orchestrated biological processes of insulin synthesis and secretion are dynamically regulated during the pathogenesis of T2D (Figure 2), within which it is clear that β-cell function can be actively influenced and affected via multiple signaling pathways. RNA sequencing (RNA-Seq) and proteomic analysis in islets isolated from GK rats (a model of T2D) revealed two distinct stages of transcriptomic and proteomic changes along with the progression of T2D (Hou et al., 2017). Correspondingly, the functional alteration of β-cells during T2D could also be divided into two major stages: compensation and decompensation. In normal glucose-tolerant individuals, there is a hyperbolic function between insulin secretion and insulin sensitivity, of which insulin-resistant individuals require increased levels of insulin to achieve normoglycemia compared to insulin-sensitive subjects. β-cells detect this requirement via glucose sensing. Under long-term high-glucose stimulation, β-cells compensate to restore glucose homeostasis by promoting β-cell hypertrophy and hyperplasia for increased insulin biosynthesis (Cerf, 2015). As observed in animal models, hyperglycemia, accompanied by obesity and insulin resistance, initially induces β-cell compensatory responses to increase insulin synthesis and secretion as well as promote β-cell proliferation and hypertrophy to increase β-cell mass (Okamoto et al., 2006; Zhang et al., 2010). Increased glucose also increases the β-cell proliferation rate *in vitro* (Kwon et al., 2004), where rodent models have demonstrated that glucose infusion results in an increased rate of β-cell proliferation (Bonner-Weir et al., 1989; Paris et al., 2003). It can therefore be inferred that glucose level is one of the key systemic factors controlling β-cell compensation (Porat et al., 2011). Also, it was shown that activation of Toll-like receptors TLR2 and TLR4, two presumed free fatty acid (FFA) sensors, blocked β-cell proliferation in mice and humans (Ji et al., 2019), suggesting a potential role of accumulating FFAs in regulating β-cell proliferation.

During T2D development, the compensation of β-cell function is always coupled with escalating insulin resistance. This results in prolonged hyperinsulinemia, which further desensitizes IRs, deteriorates insulin resistance, and increases the workload of β-cells. Together with other factors including glucotoxicity, lipotoxicity, and increasing meta-inflammation, such devastating burden will then lead to a loss of β-cell compensation and their switch to a decompensation functional stage. This represents a key negative turning point in T2D progression. With increasing time when β-cell death continues to exceed β-cell replenishment and functional β-cell mass declines, a clear progression of T2D is navigated after this turning point.

It has been claimed that β-cell compensation and decompensation mechanistically result from short-term and long-term metabolic stress, respectively. β-cell metabolic stress is a comprehensive concept mediated by hyperglycemia, hyperlipidemia, and hyperinsulinemia and is caused by excessive energy intake and insulin resistance in peripheral tissues. However, recent studies have found that the source of metabolic stress is wider than originally thought, involving organs such as the brain, liver, muscle, fat, kidney, and intestine, and is also related to the microenvironment of the islet (Defronzo, 2009). Chronic metabolic stress can cause irreversible damage to β-cells, building up ER stress (Eizirik et al., 2008), oxidative stress, and inflammation (Nordmann et al., 2017). These three pathways are not independent of each other in the process of β-cell functional alternation but intertwine as a network. The adverse outcomes of this process include apoptosis and loss of β-cell identity (dedifferentiation and transdifferentiation) (Bergman et al., 2002), which serve as immediate causes of β-cell mass reduction and damage.

### Role of ER stress in β-cell dysfunction in T2D

Pancreatic β-cells are burdened with high secretory demand of insulin and other hormones, making them particularly vulnerable to the secretory-pathway-related stress, especially under increasing insulin demands caused by insulin resistance. Increasing insulin synthesis and secretion is believed to be a direct cause of overloaded protein folding machinery and the resulting accumulation of unfolded or incorrectly folded proteins (Eizirik et al., 2008; Hetz, 2012). During the transition from compensation to decompensation, the secretion of proinsulin significantly increases, which is indicative of impaired insulin processing (Huang et al., 2019) in both the ER and Golgi apparatus. The accumulation of unfolded proteins then triggers unfolded protein responses (UPRs) and ER stress. In addition, the UPR pathway can also be activated under excessive FFAs, hyperglycemia, and increasing islet amyloid polypeptide conditions (Huang et al., 2007; Yamamoto et al., 2019). For example, palmitate induces rapid Ca^2+^-dependent degradation of carboxypeptidase E, the final enzyme required for insulin maturation, resulting in the accumulation of proinsulin (Jeffrey et al., 2008).

Previous *in vivo* and *in vitro* studies have shown that ER stress is a key factor in β-cell damage caused by high glucose levels. High insulin demand is imposed on β-cell in the presence of insulin resistance, which stimulates the synthesis of insulin and is likely to promote the misfolding of insulin (Ozcan et al., 2006). Besides, high glucose stimulation significantly increases the expression of UPR-related genes in β-cells, and excessive stimulation of the UPR pathway is linked to β-cell glucotoxicity (Bensellam et al., 2012). FFA exposure stimulates eIF2 phosphorylation, induces ATF6, C/EBP homologous protein (CHOP), and BiP expression in β-cells, suggesting its participation in β-cell ER stress (Kharroubi et al., 2004; Cnop et al., 2007). Mechanically, saturated FFAs may trigger ER stress via overburdening the ER and interrupting protein transportation from the ER to the Golgi (Preston et al., 2009). Simultaneously, the improvement in lipid metabolism brought about by high-density lipoproteins (HDLs) restores ER-to-Golgi-transportation homeostasis and relieves β-cell from ER stress (Pétremand et al., 2012).

Three main ER-resident signaling molecules, PERK, ATF6, and IRE1, act as sensors to trigger cellular responses in adaptation to ER stress (Lin et al., 2007; Hetz, 2012). Such responses include the inhibition of translation (Harding et al., 2000), the induction of mRNA degradation (Hollien and Weissman, 2006; Han et al., 2009), the activation of autophagy (Kroemer et al., 2010; B'Chir et al., 2013), the stimulation of ER biogenesis (Lee et al., 2005), an upregulation in the expression of chaperones (Huang et al., 2019), and the degrading of unfolded and misfolded proteins (Hwang and Qi, 2018). Despite these responses being short-term protective to islet β-cells, overwhelming and chronic ER stress soon begins to induce β-cell apoptosis (Hetz, 2012). Experiments on humans and mice revealed an enhanced β-cell death due to a high susceptibility to ER stress inducers in individuals with PERK mutations. CHOP, a downstream factor of PERK and ATF6, is possibly involved in the mediation of such an ER stress-induced apoptosis. Suppression of the CHOP gene in mice can alleviate ER stress (Yong et al., 2021) as well as mitigate β-cell apoptosis following ER stress. Therefore, such manipulation of CHOP helps prevent diabetes development (Eizirik et al., 2008; Song et al., 2008). Overexpression of CHOP and ATF4, its upstream regulator, has the opposite effect (Han et al., 2013). Previous studies suggest that CHOP deteriorates ER stress and accelerates cell death via promoting protein synthesis and oxidative stress (Han et al., 2013). In addition, ER stress damages β-cells possibly through altering Ca^2+^ homeostasis. It has been indicated that ER stress interferes with the function of RyR located in the membrane of the ER and causes leakage of ER Ca^2+^ (Yamamoto et al., 2019). The destruction of β-cell's ER Ca^2+^ homeostasis results in impaired insulin secretion and further promotion of β-cell death. Moreover, Lin et al. (2007) found that persistent ER stress attenuates the activity of the ATF6 and IRE1 pathways but leaves the PERK pathway intact, implying diverse roles from different stress response pathways in determining the cell fate under the pressure of ER stress. One possibility is that the β-cell transition from compensation to decompensation may correspond to an equivalent protective-to-cytotoxic transition under ER stress responses. However, systematic analysis of the temporal and causal relationship between these shifts is still lacking, and detailed mechanisms are yet to be revealed.

### Role of oxidative stress in β-cell dysfunction in T2D

In addition to ER stress, oxidative stress with characteristics of increased levels of reactive oxygen species (ROS) is also a noted factor in the pathogenesis of β-cell dysfunction in T2D (Burgos-Morón et al., 2019; Huang et al., 2019). ROS are by-products of various cellular physiological activities, which are mainly linked to mitochondrial aerobic respiration (Bae et al., 2011). In β-cells, increasing cellular glucose and FFA under hyperglycemic and hyperlipidemic conditions augments glucose and lipid oxidation along with an elevated level of ROS generation (Burgos-Morón et al., 2019). In response to a high extracellular glucose concentration, β-cells show a quick and proportional increase in glycolysis. Due to the low lactic acid metabolism in β-cells under natural conditions (Sekine et al., 1994), glucose is totally oxidized when it enters the cells. The formation of ROS in β-cells can be induced by an increase in mitochondrial oxidative metabolism, the continuous activation of Ca^2+^ channels, and hyperinsulinemia, all of which can lead to oxidative stress (Sampson et al., 2010; Rharass et al., 2014). In addition, FFAs may cause β-cell oxidative stress through a variety of pathways. On one hand, increased lipid oxidative metabolism in β-cells may result in an increase in ROS production. FFA levels overburden the mitochondria, resulting in incomplete FFA oxidation, which contributes to the increase in ROS (Burgos-Morón et al., 2019). Meanwhile, lipid-mediated cell oxidative stress could also be related to its suppressive effect on KIF12, a microtubule motor involved in β-cell antioxidant activity (Yang et al., 2014). KIF12 scaffolds Sp1, a transcription factor that appears to be essential for optimal peroxisomal function by raising Hsc70 expression, which in turn alleviates oxidative stress (Yang et al., 2014). Besides, inflammatory factors can also cause oxidative stress through modulating the Nrf2/NF-κB and SAPK/JNK pathways (Choudhury et al., 2015). In the crosstalk between inflammation and β-cell oxidative stress, divalent metal transporter 1 may play an essential role (Hansen et al., 2012).

ROS can serve as signaling molecules and play a vital part in signal transduction (Reczek and Chandel, 2015). However, an excessive amount of ROS under pathological conditions causes damage to the cellular nucleic acids, proteins, and lipids, leading to mitochondrial dysfunction or even cell death. Generally, cells have evolved to neutralize excess ROS with scavenger enzymes such as superoxide dismutase, catalase, and glutathione peroxidase. However, these antioxidant enzymes are under-expressed in β-cells, resulting in the increased sensitivity of β-cells to the threats posed by excessive ROS production. Other factors with protective roles against oxidative stress, Rheb1 and Fam3a for instance, are also found to downregulate in β-cells under diabetic conditions (Yang et al., 2020, 2021). In addition, due to the inherent vulnerability of mitochondrial DNA (mtDNA) against elevated ROS levels, oxidative stress is believed to induce lesions to mtDNA so that mitochondrial dysfunction in diabetic conditions is developed (Chow et al., 2017). ROS may also damage β-cells by interrupting mitochondrial dynamics. Research on Chang cells demonstrates that ROS suppresses Fis-1, a regulator of mitochondrial fission, and results in the generation of elongated and oversized mitochondria (Yoon et al., 2006). Combined with Yoon's results from *in vitro* experiments, observation begins to hint the importance of Fis-1 in β-cell GSIS (Schultz et al., 2016), suggesting that ROS can impair β-cell function via the disruption of Fis-1, which in turn causes aberrant mitochondrial fusion. Correspondingly, decreased mitochondrial function leads to changes in the metabolic pattern of β-cells, in which the oxidative metabolic pathway is blocked so that lactate synthesis is forced to increase (Hou et al., 2017).

Under oxidative stress, cells are still prone to protect themselves by removing or neutralizing excessive ROS. As mentioned above, promoting autophagy, upregulating the antioxidative system and its related antioxidant enzymes, and increasing reductive chaperone-like antioxidants such as resident protein disulfide isomerases, endoplasmic reticulum oxidoreductin1, and glutathione (Zuo et al., 2015; Burgos-Morón et al., 2019) are the major approaches against oxidative stress. Cells may also undergo mitophagy, by which damaged mitochondria are degraded to maintain mitochondrial homeostasis (Palikaras et al., 2018). Despite the fact that during the β-cell compensation process, sufficient antioxidants may effectively protect β-cells from ROS-related damage, the relentless level of oxidative stress created by a chronic increase in ROS once β-cells enter the decompensation phase and initiate the onset of T2D may deplete the cellular reservoir of these antioxidants (Zeeshan et al., 2016). Irreversible damage thereby occurs in the decompensation phase, including DNA damage (Dandona et al., 1996; Al-Aubaidy and Jelinek, 2011) and apoptosis, which have been suggested to be mediated directly by ROS and its downstream molecules.

### Role of apoptosis in β-cell dysfunction in T2D

As the driving force of β-cell decomposition, the apoptosis of β-cells is a topic drawing increasing attention for its significant contribution to T2D development and aspects of T2D pathology (Rhodes, 2005). It has been proposed that elevated β-cell apoptosis outweighs both reduced replication and neogenesis in terms of its overall contribution to the decline of β-cell mass in the decompensation period. Long-term exposure to FFAs inflicts human pancreatic islets with cytostatic and pro-apoptotic impacts, which are likely to be mediated by caspases and Bcl-2 via the ceramide pathway (Lupi et al., 2002). Meanwhile, the mitochondrial pathway may also play an important role in FFA-mediated β-cell apoptosis (Maedler et al., 2001). Elevated fatty acids, such as palmitate, have direct toxic effects on β-cells via activating the caspase-3-dependent mitochondrial apoptosis pathway (Jeffrey et al., 2008). The activation of caspase-3 by palmitate is synergistic with glucotoxicity (Prentki and Nolan, 2006), but its capacity in triggering β-cell apoptosis is independent of the presence or absence of elevated glucose levels (Jeffrey et al., 2008). Palmitate decreases the expression of the anti-apoptotic Bcl-2 protein (Lupi et al., 2002) and is associated with the T2D susceptibility gene, calpain-10 (Johnson et al., 2004). In the β-cell, calpain-10 likely plays a pro-apoptotic role that is independent of the promotion of insulin secretion (Marshall et al., 2005). In European populations, TCF7L2, a transcription factor involved in the development and survival of islet cells and enteroendocrine cells of the gut has been implicated in β-cell apoptosis accompanied with increased caspase-3 cleavage and decreased AKT activity (Shu et al., 2008). Pancreatic β-cell function is also reduced in patients with TCF7L2 polymorphisms (Lyssenko et al., 2008).

### Role of differentiation and reprogramming in β-cell dysfunction in T2D

Parallel to the apoptotic mechanism, new research has revealed that β-cells can also shed their identities and undergo dedifferentiation or transdifferentiation in response to metabolic stimuli. It has been established that β-cell can be converted into a progenitor-cell-like form after the inactivation of certain identity transcription factors (Talchai et al., 2012). This process is therefore named dedifferentiation as in reverse chronological order to normal cellular differentiation. Studies conducted on mice have revealed that dedifferentiation also contributes to diabetic β-cell failure. Lineage-tracing experiments have even suggested that dedifferentiation may play a major role in the process of mouse β-cell loss (Talchai et al., 2012). A similar phenomenon has been also observed in human β-cells, of which ∼31.9% are dedifferentiated in T2D patients compared with only 8.7% dedifferentiated in normal individuals (Cinti et al., 2016). The discovery of dedifferentiation reminds us that it is the number of specifically functional β-cells but not the total number of all types of β-cells that requires consideration in the research of β-cell failure.

Mature β-cells are featured by several transcription factors that are indispensable in the regulation of the synthesis and secretion of insulin, such as FOXO1, PDX1, NKX6-1, NEUROD1, and MAFA. Dysregulation of these genes can result in β-cell dedifferentiation (Guo et al., 2013). Conversely, ectopic expression of these genes in the intestine, stomach, liver, pancreas, and gallbladder can reprogram these organs to produce insulin (McKimpson and Accili, 2019). Among these β-cell identity-maintaining genes, the role of FOXO1 has been well studied. FOXO1 is the upstream regulator of NEUROD1 and MAFA (Kitamura et al., 2005). Under oxidative stress conditions, FOXO1 is translocated into the nucleus (Furukawa-Hibi et al., 2005) and upregulates the expression of these two genes. However, translocation of FOXO1 is impaired in the face of relentless metabolic stress, and depletion of FOXO1 is observed in β-cells that have been exposed to chronic metabolic stress (Talchai et al., 2012). It is also worth noting that the abnormal expression of progenitor-specific genes or disallowed genes (Lemaire et al., 2016), such as NANOG and MYCL in β-cells, also induces dedifferentiation (Talchai et al., 2012). This leads to the suggestion that dedifferentiation is probably also a result of the failure to suppress disallowed genes. Thus, importance should be attached to the search for ‘suppressor genes’. Lu et al. (2018) reported that the depletion of the polycomb complex PRC2, an epigenetic repressor responsible for global gene silencing, leads to the upregulation of genes specific to immature and progenitor β-cells. This upregulation induced by PRC2 depletion corresponds to the observation that β-cells from T2D individuals and in mice with a high-fat diet (HFD) tend to have relatively active transcription at the target loci of PRC2. Loss of PRC2 upregulates Gil2 (Lu et al., 2018), suggesting that PRC2 dysfunction may induce β-cell dedifferentiation via the Hedgehog/Gli signaling pathway (Landsman et al., 2011). Also, other potential factors like Grb10, which is highly expressed in diabetic β-cells, were recently shown to play important roles in regulating β-cell proliferation and dedifferentiation (Cai et al., 2022).

Aside from dedifferentiation, lentiviral-mediated lineage tracing and observation of islets from individuals with T2D discovered a process of transdifferentiation, in which β-cells can be converted into other islet cell types, such as glucagon-producing α-cells (Spijker et al., 2013; Cinti et al., 2016) and somatostatin-producing δ-cells (Cinti et al., 2016). Transdifferentiation is probably induced by the aberrant gene expression of ARX and PAX4, which are in pairs antagonizing each other during the differentiation of progenitor pancreatic endocrine cells (Collombat et al., 2003). Whilst ARX induces differentiation toward α-cells (Courtney et al., 2013), PAX4 is necessary for the process of progenitors acquiring a β-cell identity (Sosa-Pineda et al., 1997). In natural conditions, further research is required to determine whether the transdifferentiation of β-cells to α-cells needs to first undergo a dedifferentiation process backward to the progenitor-cell phase. Some authors have assumed that transdifferentiation occurs after differentiation. However, cells secreting both insulin and glucagon may be found in the islets of T2D patients, implying the existence of a more direct transdifferentiation process between these two types of cells. Therefore, transdifferentiation can be a possible mechanism of β-cell decompensation. Further research is required to clarify whether transdifferentiation could be a cause or a result of T2D (Eizirik et al., 2020).

Blood glucose levels regulate insulin production at both transcriptional and post-transcriptional stages. The regulation could be mediated by the modulation of the transcription factors PDX1, NEUROD1, and MAFA, and the processes through which glucose affects the function of the three transcription factors can be different (Andrali et al., 2008). Reduction in insulin gene expression has been found in β-cell lines and islets isolated from rodents and human islets after prolonged exposure to high glucose levels, which is one of the most well-studied alterations related to glucotoxicity (Robertson et al., 1992; Briaud et al., 1999; Marshak et al., 1999). This has been associated with reduced expression and binding of transcription factors to the insulin gene promoter, such as PDX1 and MAFA (Poitout et al., 1996; Moran et al., 1997; Marshak et al., 1999; Harmon et al., 2005). From the perspective of β-cell differentiation and reprogramming, the regulation of these transcription factors by glucose may provide a possible explanation for the mechanism of dedifferentiation and transdifferentiation of β-cells under the condition of synchronous high glucose.

### Islet microenvironment and immune response in the pathogenesis of β-cell dysfunction in T2D

To investigate changes in β-cell function, we must consider both the internal physiological activities of β-cells under metabolic stress and the macroscopical aspects such as β-cell population changes, interactions with other cells, and cells in the islet microenvironment. The overall β-cell mass is dynamic and able to respond to metabolic demands by mechanisms of compensation and replenishment from various cell sources, which have different capacities depending on their metabolic states. Conversely, inadequate β-cell replenishment and failure to sustain β-cell compensation clearly lead to the onset of β-cell dysfunction. The compensatory capacity of β-cells may be related to their ability to proliferate. The ability of islet β-cells to proliferate has been shown to be reduced in aged rodents (Finegood et al., 1995) and humans (Perl et al., 2010), which links to the corresponding observation of an increasing incidence of T2D with age. In addition, the incidence of insulin-positive neogenic but non-proliferative cells was seen to be increased during the compensation phase, suggesting that non-proliferative β-cell growth is one mechanism of β-cell mass compensation (Huang et al., 2019). Decreased neogenesis and β-cell dedifferentiation were correspondingly observed in β-cell dysfunction (Huang et al., 2019). In this sense, activation of genes involved in β-cell proliferation and regeneration could be a promising approach for preventing β-cell decompensation.

The islet microenvironment also has an effect on β-cells. Under normal conditions, β-cells need to interact with other types of islet cells and endothelial cells to together comprise the microcirculation within islets and neurons. Such interactions help conduct neurological control of islet β-cells and thus sustain their normal functions (Brissova et al., 2006; Thorens, 2011). A change in the β-cell population in the pancreas will therefore affect other islet cell populations due to the local effects of other islet hormones on insulin. As a result, upon β-cell restoration, other islet cells must also be replenished in order to maintain glucose homeostasis. Following islet cell population replenishment, the islets are then reset to meet the current metabolic demand. It has been reported that alternation of the islet microenvironment by miR-7 is coupled with β-cell dedifferentiation (de Jesus et al., 2021). However, the specific mechanism of interaction between islet β-cells and the islet microenvironment requires further study to fully unravel.

Mechanisms underlying the β-cell compensation-to-decompensation switch are also related to immune responses. In general, inflammation is much more severe in the decompensation phase. Diabetic islets are characterized by their inflammatory features, including increased macrophage infiltration and increased release of inflammatory factors (Ehses et al., 2007; Böni-Schnetzler et al., 2008). Islet inflammatory cytokines such as interleukin-1 (IL-1), TNFα, and IFNγ can act via nitric oxide to decrease the expression of the SERCA pumps that load Ca^2+^ into the ER (Cardozo et al., 2005). Although the stimulation of inflammatory cytokines can increase the concentration of cytoplasmic Ca^2+^ by preventing ER Ca^2+^ loading and thus promote insulin secretion in the short term, chronic exposure to these inflammatory cytokines depletes ER Ca^2+^ storage and results in impaired insulin secretion (Cardozo et al., 2005). Moreover, Ca^2+^-dependent protein processing and β-cell apoptosis are also promoted in such circumstances (Eizirik et al., 2008). Another study in 2015 suggests that inflammation induces ROS production and promotes β-cell death via Nrf2/NF-κB and SAPK/JNK pathways (Choudhury et al., 2015). These studies show that the cellular responses triggered by proinflammatory cytokines are closely related to ER stress and oxidative stress. Taken together, these researches further substantiate the significance of the fine-tuned immune microenvironment in regulating β-cell function.

Overnutrition, on one hand, stimulates peripheral tissues such as the adipose tissue, muscle, and liver to release inflammatory factors into the circulation, causing islet inflammation further (Donath and Shoelson, 2011). On the other hand, overnutrition-related hyperglycemia and hyperlipidemia have been suggested to have a direct effect on the islets and trigger an inflammatory response. According to Eguchi et al. (2012), palmitate, the most abundant saturated FFA in blood, causes β-cell dysfunction *in vivo* by triggering inflammatory processes inside islets through the TLR4/MyD88 pathway. In mice and rats, inhibiting the proinflammatory kinase IKK reduces FFA-induced β-cell dysfunction (Ivovic et al., 2017). Inflammation activates oxidative stress, and the latter, in turn, is reported to stimulate the NF-κB pathway, which escalates β-cell inflammation (Morgan and Liu, 2011). Furthermore, saturated fatty acids (SFAs) have been shown to activate NLRP3–ASC inflammasomes, reducing insulin sensitivity via the IL-1- and/or TNF-inflammatory pathways (Wen et al., 2011). However, whether this pathway affects β-cell insulin sensitivity remains to be established.

### Emerging role of the cGAS–STING pathway in β-cell dysfunction in T2D

Inflammatory responses in β-cells have also been associated with the islet microenvironment and immune–endocrine cell communications and interactions. Notably, the activation of the cyclic guanosine monophosphate–AMP synthase (cGAS)–stimulator of interferon genes (STING) pathway has been reported as a key molecular link between immunity and metabolism in multiple metabolic diseases (Bai and Liu, 2019, 2021). Remarkably, a previous study revealed that HFD-induced mtDNA release into the cytosol of adipocytes triggers the activation of the cGAS–STING pathway and inflammatory response, leading to chronic inflammation in adipose tissue and insulin resistance (Bai et al., 2017). The mitochondrial stress-activated cGAS–STING pathway has also been found to suppress thermogenesis in adipose tissue, thereby contributing to overnutrition-induced obesity (Bai et al., 2020). A previous study demonstrated that activation of STING–IRF3 in hepatocytes contributes to hepatic inflammation and apoptosis in HFD-induced nonalcoholic fatty liver disease in mice (Qiao et al., 2018). Moreover, STING induces inflammation in nonalcoholic steatohepatitis pathogenesis as a proposed mtDNA sensor in Kupffer cells of the liver (Yu et al., 2019). In pancreatic β-cells, Hu et al. (2020) reported that the activation of STING–IRF3 signaling plays an important role in mediating the effects of lipotoxicity on pancreatic β-cell inflammation and apoptosis. More recently, Gao et al. (2022) showed that intestinal microbial DNA-containing extracellular vesicles could pass obese gut barrier and deliver microbial DNA into β-cells to trigger cGAS–STING activation, resulting in elevated inflammation and impaired insulin secretion. This work adds to our understanding of how the islet microenvironment interacts with gut microorganisms. The processes driving extracellular vesicles to translocate from the gastrointestinal lumen into the human circulation and distant tissues, on the other hand, are yet to be explored. These findings imply that, other than immune cells, β-cells themselves might also actively contribute to the formation and regulation of the immune microenvironment in pancreatic islets by expressing cytokines, which at least are partially mediated through the cGAS–STING signaling pathway. Considering the accumulating ER stress and oxidative stress in islet β-cells during T2D pathogenesis as discussed above, one could also reason that another trigger of the cGAS–STING pathway in β-cells could be attributed to stress-induced mtDNA release, which thereby contributes to the formation of a proinflammatory microenvironment in the islet and to the loss of GSIS function in β-cells. Evidence is yet to be discovered to confirm such a hypothesis.

More interestingly, our recent study revealed a distinct role of STING in insulin action regulation in peripheral metabolic tissues and insulin secretion from β-cells (Qiao et al., 2022). STING expression is significantly downregulated in islets from diabetic *db/db* mice and human T2D patients. Although whole-body knockout of STING in mice exhibited an improvement in insulin sensitivity and glucose homeostasis responding to HFD feeding, β-cell-specific inactivation of STING impaired its GSIS function and systemic glucose intolerance. Mechanistically, we demonstrated that STING ablation gave rise to a decrease in transcription, nuclear localization, and binding activity of PAX6 in which lower binding activity to the promoter regions of its target genes led to downregulation of genes important for β-cell glucose uptake and insulin secretion, such as GLUT2, Abcc8, and Kcnj11. Combining our new findings with previous studies, the importance of fine-tuned STING signaling pathway activity in β-cells and insulin-targeting metabolic tissues in controlling whole-body glucose homeostasis is emphasized. These data also point to the requirement of tissue-specific STING-targeting strategies for T2D treatment.

### Role of impaired insulin signaling in β-cell dysfunction in T2D

The compromised insulin signaling pathway and inadequate capacity to respond to insulin in islets can be referred to as ‘β-cell insulin resistance’, similar to insulin resistance in peripheral tissues. β-cell-specific IR deletion results in impaired glucose tolerance (Kulkarni et al., 1999) and failure in β-cell compensatory proliferation (Okada et al., 2007). Meanwhile, FOXO1 nucleates and PDX1 expression declines, implying that insulin signaling pathway dysfunction is linked to FOXO1 and PDX1 loss-of-function (Okada et al., 2007). Lipotoxicity, in combination with islet inflammation, is likely to cause β-cell insulin resistance (Zheng et al., 2022). The degradation of β-cell function may be partially attributed to a defect in insulin signaling caused by prolonged metabolic stress in T2D, but the fundamental processes are still incompletely elucidated.

### Role of mTOR signaling in β-cell dysfunction in T2D

The mTOR signaling system detects nutrition, energy, stress, and growth stimuli in mammals and links nutrient and energy availability to cell growth and division (Saxton and Sabatini, 2017). According to the subunit constitution, mTOR protein complexes can be classified into two types, known as mTORC1 and mTORC2. In β-cells, these two complexes may have different functions. mTOR signaling regulates major β-cell biological functions such as cell growth, proliferation, and insulin secretion; dysregulated mTOR signaling, instead, leads to β-cell dysfunction and death.

mTORC1 activity is influenced by a variety of upstream signals. Increased mTORC1 signaling is seen in obese or HFD-treated animals (Khamzina et al., 2005), β-cells of *db/db* mice (Jaafar et al., 2019), and diabetic islets (Yuan et al., 2017a), which is likely owing to the stimulation of insulin, amino acids, glucose, and proinflammatory cytokines. According to studies on mTORC1-hyperactivated mice, the effect of mTORC1 on β-cell activity appears to be biphasic (Shigeyama et al., 2008). With hyperactivated mTORC1, juvenile β-cell-TSC2KO mice have more β-cell mass, higher insulin levels, and better glucose tolerance. This effect, however, is reversed in older β-cell-TSC2KO mice that have less β-cell mass, lower insulin levels, and hyperglycemia (Shigeyama et al., 2008; Mori et al., 2009). Despite the importance of mTORC1 in the differentiation and proliferation of immature β-cells (Ding et al., 2017), the partial suppression of mTORC1 in β-cells may be coupled with the maturation of β-cells, and the abnormal hyperactivation of mTORC1 under pathological conditions such as malnutrition and hyperinsulinemia may lead to the transformation of β-cells into dysfunctional immature precursors (Jaafar et al., 2019), suggesting the involvement of mTORC1 in β-cell identity loss. Considering the similar β-cell behavior of mTORC1-hyperactivated mice and HFD mice, the reactivation of mTORC1 is likely to play a role in the dynamic alternations of β-cells under metabolic stress. The involvement of mTORC1 in the transition from compensation to compensation, however, needs to be further studied.

The phosphorylation and stimulation of AKT, a major regulator of insulin/PI3K signaling, is likely a critical function of mTORC2 (Sarbassov et al., 2005). In contrast to mTORC1, the function of mTORC2 is downregulated in islets from T2D patients, as well as the islets and β-cell lines under the stress of high glucose concentrations (Yuan et al., 2017a). Disabling the mTORC2 complex using β-cell-specific Rictor knockout results in reduced β-cell mass, proliferation, insulin content, and GSIS (Gu et al., 2011). The phenotype was enhanced under high-fat feeding, suggesting that mTORC2 may play an important role in the compensatory response of β-cell to metabolic stress, possibly through the phosphorylation of PKCα (Xie et al., 2017). Considering the intersection of mTORC2 and insulin downstream signaling pathway, targeting the β-cell mTORC2 signaling pathway may have therapeutic significance for β-cell dysfunction caused by insulin signaling pathway disorder.

### Role of Notch signaling in β-cell dysfunction in T2D

Notch, an evolutionary-conserved signaling pathway, dictates cell fate through juxtacrine interactions between adjacent cells expressing Notch receptors and ligands (Artavanis-Tsakonas et al., 1999). Notch signaling pathway plays a key role in modulating pancreatic embryonic development and pancreatic cell differentiation, including preventing the differentiation of premature pancreatic endocrine and ductal cells (Apelqvist et al., 1999; Fujikura et al., 2006). However, the signaling pathway is largely suppressed in mature β-cells (Bartolome et al., 2019). The β-cell-specific Notch knockout results in improved glucose tolerance when subjected to HFD feeding. Conversely, β-cell-specific Notch gain-of-function manifest deteriorates β-cell identity loss (Bartolome et al., 2019). This is consistent with the findings that isolated pancreatic islets from diabetic NOD mice have elevated expression of critical Notch signaling genes (Billiard et al., 2018). In this sense, the abnormal reactivation of Notch is a possible way of β-cell dedifferentiation under diabetic conditions. However, the mechanism of Notch being reactivated in mature β-cell is not completely clear, and the role of metabolic stress in this process remains to be explored.

## Effects of dietary interventions on β-cell function

**Figure 3:**
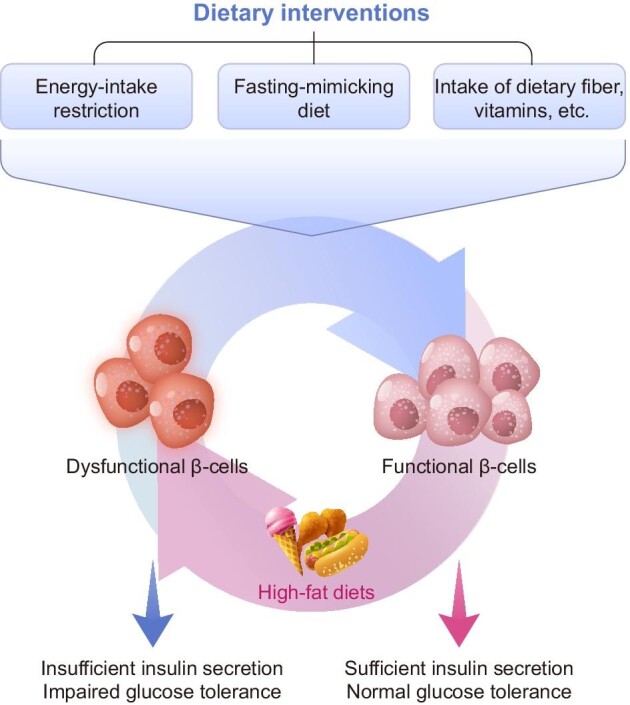
Schematic diagram of dietary intervention-mediated regulation of insulin secretion in β-cells. Three widely established dietary intervention strategies for sustaining β-cell function and enhancing glycemic control are restricting calorie intake, following a fasting-like diet, and altering the intake of specific dietary components. Alleviating metabolic stress, maintaining β-cell mass, promoting insulin secretion, and preserving β-cell identity are all possible mechanisms for dietary interventions to protect β-cell function.

Dietary intervention has long been considered an effective approach to controlling the pathogenesis of T2D (Ley et al., 2014). In general, (i) limiting overall energy intake, (ii) switching to a fasting-mimicking diet, and (iii) altering the intake of specific dietary factors have been three widely applied dietary intervention approaches to preserving β-cell function and improving glycemic control. Possible pathways for dietary treatments to safeguard β-cell function include reducing metabolic stress, boosting cell survival and proliferation, promoting insulin secretion, and reversing dedifferentiation and transdifferentiation (Figure 3). The following sections will review the recognized dietary intervention mechanisms that protect β-cells.

Since T2D is tightly linked to overnutrition, limiting energy intake is considered a natural solution to prevent T2D. The conspicuous outcome of restricting energy intake is the loss of body weight. It has been reported that in humans with obesity, a moderate 5% weight loss contributes to and corresponds with improved β-cell function and improved insulin sensitivity in the adipose tissue, liver, and muscle. With additional weight loss, the trend is further confirmed (Magkos et al., 2016). In addition, recovery of both β-cell function and hepatic insulin sensitivity in T2D can be achieved by reducing dietary energy intake alone, associated with decreased pancreatic triacylglycerol stores (Lim et al., 2011). Meanwhile, ameliorated metabolic disorders are also observed upon intermittent fasting, defined as periods of voluntary fasting from food and drink (de Cabo and Mattson, 2019). The intermittent fasting approach does not entail a long-term adjustment in total calorie intake, implying that the effect of fasting extends beyond simply lowering total energy consumption (Anson et al., 2003). The possible role of intermittent fasting in improving metabolism suggests that fasting at a particular time length may trigger metallic switching mechanisms protective to β-cells. However, a recent clinical study shows that intermittent fasting does not significantly improve metabolism on the basis of calorie restriction (Liu et al., 2022), suggesting that the benefits of intermittent fasting may largely be due to calorie restriction.

In addition to a fasting strategy, adjusting the dietary nutrient composition, in other words, changing the intake of various dietary components, is also an important aspect of diabetic dietary intervention. Some widely applied diets for controlling diabetes, such as the Mediterranean diet, emphasize altering the intake amount of specific dietary factors, including lipids, dietary fiber, vitamins, etc. Abundant clinical studies have shown that dietary factors can have a variety of effects on diabetes, some of which are well known. The effects of these dietary factors on β-cells, as well as their possible molecular mechanisms, are discussed below.

### Effect of dietary interventions on β-cell metabolic stress

As discussed above, excessive dietary caloric consumption results in increased insulin demand, as well as glucotoxicity and lipotoxicity, causing ER stress, oxidative stress, and inflammation, all of which could be linked to β-cell dysfunction. The drivers of β-cell functional alternations as described above, ER stress, oxidative stress, inflammation, etc., are observed to be suppressed in diabetic mice receiving dietary restriction (Kanda et al., 2015; de Cabo and Mattson, 2019). Practically, reducing insulin demand and lowering hyperglycemia and hyperlipidemia, known as the therapy of ‘resting’ β-cells, have been shown to be effective means of restoring insulin secretion function (Marshak et al., 1999; Boland et al., 2019).

Besides, increasing the intake of antioxidants is a conceivable therapy for preventing or treating β-cell dysfunction given the significance of oxidative stress in the process. Indeed, a vast number of studies have documented the benefits of antioxidant-rich diets in the prevention and treatment of diabetes (Burgos-Morón et al., 2019). Improving β-cell tolerance to oxidative stress may explain the therapeutic benefits of dietary antioxidants, such as vitamins and polyphenols, in this respect (Burgos-Morón et al., 2019). Nevertheless, the impact of some dietary antioxidants on β-cell oxidative stress has to be further validated. Besides, dietary quercetin and carnosine may also have antioxidant properties. Quercetin, a flavanol compound enriched in vegetables and fruits, is reported to inhibit β-cell oxidative stress via the ERK1/2 pathway (Youl et al., 2010). Carnosine, a dipeptide consisting of alanine and histidine found largely in red meat, has the capacity to scavenge oxidizing species and has been proven in cellular investigations to result in improved insulin production and glucose uptake (Cripps et al., 2017). In addition, according to a recent study, carnosine has a strong cytoprotective impact on β-cell lines and human islets via lowering oxidative stress (Miceli et al., 2018).

Specific dietary factors, such as vitamin D and resveratrol, also protect β-cells from the destruction of islet inflammation. Vitamin D binds to the vitamin D receptor (VDR) on β-cells, causing VDR to bind with the chromatin remodeling complex PBAF, resulting in genome-wide alterations in chromatin accessibility and enhancer landscape and inducing an anti-inflammatory response (Wei et al., 2018b). Strong evidence has shown that resveratrol, a polyphenol compound rich in fruits such as grapes, exerts a protective effect on β-cells, possibly via an anti-inflammatory manner. Resveratrol protects β-cell from inflammatory cytokine damage by activating SIRT1, a NAD-dependent protein deacetylase that interferes with the NF-κB signaling pathway (Lee et al., 2009).

Furthermore, dietary interventions trigger the release of secretory factors in peripheral tissues, which may play a part in ameliorating β-cell metabolic stress. FGF21, a liver-derived secretory protein in response to fasting or a vegan diet (Byun et al., 2020), has a wide range of impacts across the body, and its effect on reducing ER stress, oxidative stress, and inflammation in diverse organs and tissues has been shown in several investigations (Fisher and Maratos-Flier, 2016). However, there are relatively few studies on the effects of FGF21 on β-cells. FGF21 has been shown to protect islets against inflammation and hyperplasia caused by an HFD (Singhal et al., 2016). Still, more studies are required to elucidate the role of FGF21 in reducing β-cell metabolic stress, and the molecular processes behind its activity in islets need to be investigated further.

### Effect of dietary interventions on β-cell insulin secretion

Considering both chronic high FFA and glucose levels are inducers of β-cell metabolic stress, the decrease in calorie intake alleviates metabolic stresses that cause β-cell GSIS destruction (Marshak et al., 1999; Boland et al., 2019). Other than FFA, cholesterol homeostasis may also play a part in β-cell function. The functional deficit of ABCA1, a cholesterol transporter, leads to β-cell dysfunction (Brunham et al., 2007). On the other hand, cholesterol accumulation in β-cells resulting from incubating cells in cholesterol solution (Hao et al., 2007) or by activating SREBP-2 (Ishikawa et al., 2008) seems to result in deterioration to the insulin secretory function.

Contrary to the toxicity of glucose and FFAs to β-cells, dietary fiber intake can protect and promote insulin secretion by affecting the intestinal microbiome, promoting the generation of short-chain-fatty-acid (SCFA)-producing strains, and promoting the production of GLP-1 (Zhao et al., 2018), which stimulates insulin secretion (Muller et al., 2019). On the contrary, a deficiency in SCFA production is associated with T2D (Qin et al., 2012). Therefore, increasing the intake of dietary fiber may be a feasible approach to protecting the secretory function of β-cells.

Moreover, our recent findings suggest that dietary intervention affects β-cell CTCF expression, triggering transcriptional reprogramming that preserves β-cell GSIS function (Wang et al., 2022). We demonstrate that CTCF expression is controlled by lipid toxicity and inflammatory signals and serves as a signal transducer incorporating these metabolic cues to the transcription of multiple GSIS-related genes, such as SLC2A2. We also reveal that CTCF is likely to mediate the protective role of dietary intervention against β-cell oxidative stress and ER stress.

### Effect of dietary interventions on β-cell survival and proliferation

As discussed above, insulin resistance and hyperglycemia lead to ER stress, oxidative stress, and inflammation, all of which are well recognized to induce β-cell death (Prentki and Nolan, 2006; Han et al., 2013). Dietary interventions targeting these metabolic stresses include limiting energy intake, maintaining β-cell mass, and inhibiting β-cell apoptosis (Kanda et al., 2015). In addition, changing the composition of food nutrients, such as replacing SFA in food with unsaturated fatty acid (USFA), has a protective effect on β-cell mass. There is evidence that, unlike SFAs, USFAs may not have lipotoxicity to β-cells and do not trigger the apoptotic process (Acosta-Montano and Garcia-Gonzalez, 2018). Interestingly, USFAs may even act as β-cell proliferation stimulators (Maedler et al., 2001). The different effects of SFAs and USFAs can be at least partly attributed to the different ways in which they are metabolized in the body. The absorption of SFA is accompanied by chylomicron, very low-density lipoprotein (VLDL), and LDL formation, while HDL is formed after the absorption of USFA. Oxidized LDL, which is increased in T2D, results in reduced proinsulin expression and JNK-mediated β-cell apoptosis, while HDL counteracts this adverse effect, possibly by inhibiting caspase-3 incision and activating AKT/PKB (Abderrahmani et al., 2007). In this sense, altering the composition of fatty acids is a means of dietary intervention, and increasing the intake of USFAs and reducing the intake of SFAs may be effective ways to reserve the β-cell mass.

Intermittent feeding can promote the proliferation of β-cells, which may be mediated by activating the autophagy–lysosome pathway and promoting the expression of Ngn3 (NEUROG3 in humans), a hallmark of pancreatic regeneration. Eight weeks of an intermittent-fasting diet turns out to be protective against β-cell mass decline (Wei et al., 2018a). Cheng et al. (2017) proved that in mice, even a 4-day feeding of intermittent fasting induces a stepwise expression of Sox17 and PDX1, followed by Ngn3-mediated generation of insulin-producing β-cells and restored insulin generation in islets. Notwithstanding sustained high-fat consumption, intermittent fasting promotes β-cell survival and nuclear expression of Ngn3 (Liu et al., 2017a). On the other hand, the inhibition of β-cell mortality and the promotion of Ngn3 expression do not occur in obese mice with lysosomal deficiency or autophagosome dysfunctions, suggesting the importance of the autophagy–lysosome pathway in this process (Liu et al., 2017a). Besides, fasting-and-diet-induced FGF21 also plays a role in promoting β-cell survival and protecting isolated rat islets and insulin-producing INS-1E cells from glucolipotoxicity and cytokine-induced apoptosis by activating the ERK1/2 and AKT signaling pathways (Wente et al., 2006).

### Effect of dietary interventions on β-cell identity loss

Metabolic stress is known to downregulate islet β-cell-specific transcription factors, such as PDX1 and MAFA, and this downregulation can hopefully be reversed by dietary restriction (Kanda et al., 2015). Although differentiation state alternations are suggested, it is unclear whether, and to what extent, β-cell dedifferentiation and transdifferentiation are involved. Emerging evidence suggests that intermittent fasting likely participates in maintaining β-cell identity by simulating Ngn3. Ngn3 determines, probably through the Notch pathway, whether progenitor cells differentiate into endocrine cells or exocrine cells (Apelqvist et al., 1999). Recently, intermittent fasting is shown to alternate pancreatic islet structure via the rearrangement of α-cell and β-cell masses and proportions (Marinho et al., 2020). By inference, intermittent fasting may reprogram islet cells and divert the direction of pancreatic cell differentiation, most likely by inducing the precursors of exocrine cells to differentiate into β-cells instead. Further studies are needed to clarify the molecular mechanism of dietary intervention on the maintenance of β-cell identity.

## Effects of exercise on β-cell function

As another important aspect of lifestyle intervention, physical exercise has been widely accepted to reduce hyperglycemia and relieve the symptoms of diabetes (Dunstan, 2008; Myers et al., 2013; Mendes et al., 2016). In the corner of pancreatic β-cells, clinical evidence has also revealed the potential of exercise in promoting β-cell function (Nieuwoudt et al., 2017; Das et al., 2018). Besides, mouse studies have demonstrated a recovered islet morphology and an increased β-cells number, both of which are in turn accompanied by reduced β-cell apoptosis upon physical exercise. The β-cell hypertrophy caused by hyperglycemia was also reversed (Medina-Contreras et al., 2017). However, the mechanism by which exercise regulates β-cell activity remains incompletely understood.

**Figure 4:**
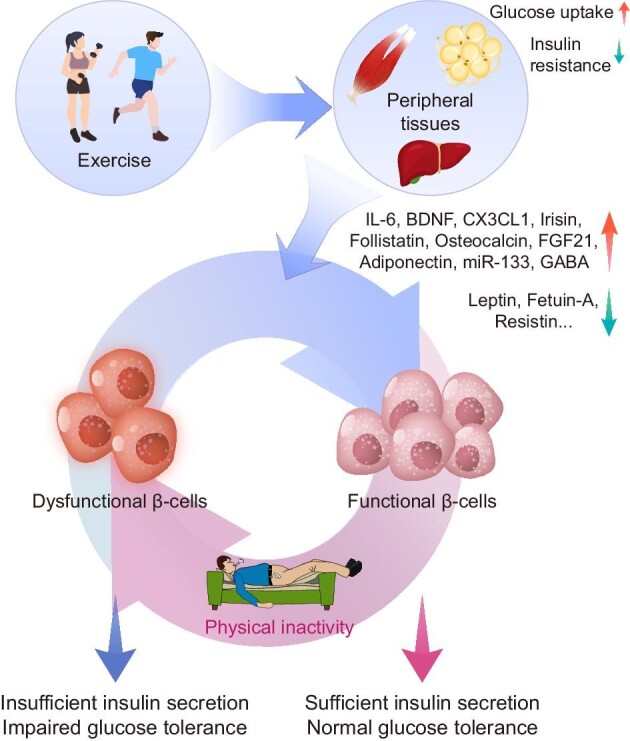
Schematic diagram of physical exercise-medicated regulation of insulin secretion in β-cells. β-cells Cells are primarily affected by exercise via the alternation of the metabolic state of peripheral tissues. On the one hand, exercise improves overnutrition by increasing peripheral tissue (e.g. the skeletal muscle, adipose tissue, and liver) glucose uptake and ameliorating insulin resistance; on the other hand, peripheral tissues under the effect of exercise modulate the secretion of a range of exerkines, which have a variety of protective effects on β-cells.

The widely accepted metabolic benefits of physical exercise on peripheral tissues, such as the muscle and liver, might be a key reason for the observed β-cell changes. Physical exercise stimulates glucose uptake in peripheral tissues, reduces peripheral insulin resistance, lowers blood glucose levels, and might hence reduce β-cell metabolic stress. The protective effects of exercise can also be attributed to endocrinologic changes in various metabolic tissues and organs in the context of ‘exerkines’ (Chow et al., 2022). Some exerkines are crucial in the alleviation of insulin resistance and glycemia by cross-talking with other peripheral tissues, and the underlying mechanisms of such process have been reviewed (Gonzalez-Gil and Elizondo-Montemayor, 2020). It is worth noticing that these multi-tissue-originated secretion factors have been generally affirmed to affect β-cells by regulating insulin secretion, β-cell proliferation, and cellular stress. Other than the contribution of peripheral tissues, neural circuits might also play important roles in mediating the protective effect on β-cells (Yamamoto et al., 2017). However, whether exercise directly affects β-cells via neural pathways remains debatable. In the following sections, we mainly discuss the beneficial effect of physical exercise on β-cells in terms of modulating ER stress, oxidative stress, and inflammation (Figure 4).

### Physical exercise relieves β-cell metabolic stress

Systematically, physical exercise increases overall energy expenditure and in turn alleviates β-cells from the metabolic stress triggered by overnutrition, noting that muscular energy expenditure is especially influenced. Exercise allows muscles to uptake glucose in an insulin-independent manner (Sylow et al., 2017), highlighting their capacity to ameliorate hyperglycemia even in the condition of insulin deficiency or resistance. The remission of hyperglycemia and peripheral insulin resistance, on the one hand, results in a decreased insulin demand, which could relieve β-cells from overloaded insulin production and reduce ER stress. In addition, reduced glucose entry into β-cells for aerobic metabolism may directly reduce ROS production and alleviate oxidative stress. Meanwhile, multiple exerkines have also been proposed to regulate β-cell metabolic stress.

ER stress and oxidative stress are major factors driving β-cell loss-of-function. Exercise intervention is suggested to protect human and rodent β-cells against ER stress (Paula et al., 2018). Downregulation of ER-stress-associated genes has been observed in the islets of resistance-exercise-trained mice (Bronczek et al., 2021). Meanwhile, exercise-trained mouse serum reduced the damage caused by CPA, an ER stress inducer, on INS-1E cell function, implying that circulating exerkine may play a role in β-cell ER stress relief (Bronczek et al., 2021). However, an exerkine that directly targets ER stress in β-cells has not yet been found. In terms of oxidative stress, it has been established in muscle tissue that ROS produced by moderate exercise can trigger a significant antioxidant stress response, which in turn eliminates ROS and prevents oxidative stress (Ristow et al., 2009). However, evidence showing that the occurrence of a similar mechanism in β-cells is insufficient. On the contrary, other ROS clearance pathways such as uncoupling protein (UCP) pathways have been reported to be present in β-cells. Because of the high sensitivity to the protonmotive force of UCP, ROS production activates UCPs and reduces the protonmotive force, which in turn decreases ROS production (Brand and Esteves, 2005). Such a negative feedback loop, therefore, protects the cell from oxidative stress. Given the role of ROS signaling in insulin secretion, UCP2 may blunt GSIS. However, its absence would be more unbearable on the other hand, since the long-term loss of UCP2 may lead to ROS accumulation, which triggers oxidative stress and damages β-cell function (Brand and Esteves, 2005). In other tissues, exercise has been reported to play a part in alleviating oxidative stress by mitigating UCP2 deficiency (Hong et al., 2021). In addition, miR-133, a muscle-derived, exercise-regulated non-coding RNA (Denham and Prestes, 2016), may be a potential regulator of this process (Chen et al., 2009). Still, the mechanism of UCP2 regulation in β-cells and the role of exercise in this process are unclear and require further elucidation. Furthermore, there is evidence that exerkines such as FGF21 (Planavila et al., 2015) and Apelin (Than et al., 2014; Vinel et al., 2018) can serve as oxidative stress inhibitors. However, whether they can ameliorate oxidative stress in β-cells has yet to be determined.

As discovered here, the impact of exercise on β-cells is largely based on its anti-inflammatory properties, which are derived from the endocrinological alternations of various metabolic tissues and organs during exercise. Both animal and human studies have found that physical exercise can reduce the level of inflammatory factors in the body and effectively reverse the body's inflammatory state (Sharif et al., 2018). The production and secretion of IL-6, the first identified and most studied myokine, can be dramatically stimulated by exercise with up to 100-fold increased circulating level (Pedersen and Febbraio, 2008). The earliest known β-cell-protective function of IL-6 is to suppress the inflammatory response. On the one hand, IL-6 antagonizes inflammatory cytokines such as IL-1 and TNFα (Schindler et al., 1990), and on the other hand, IL-6 upregulates other anti-inflammatory factors including IL-1ra and IL-10 (Steensberg et al., 2003). Recently, angiopoietin and osteoprotegerin, as two triceps-specific myokines, have also been found to have anti-inflammatory effects (Rutti et al., 2018). However, there is not enough evidence to confirm the exercise specificity of the modulation on the secretion of these two factors.

Adiponectin is an adipokine, which possesses anti-inflammatory properties and is virtually exclusively secreted by adipocytes in both white and brown adipose tissues. According to comprehensive evaluations of randomized controlled trials, exercise may boost adiponectin levels (Balducci et al., 2010; Becic et al., 2018). Adiponectin is reported to exhibit anti-inflammatory characteristics in macrophages, endothelial cells, cardiomyocytes, and fibroblasts (Ohashi et al., 2014). Adiponectin lowers TNFα expression in macrophages, suppresses macrophage-like cell response via the TLR pathway, and promotes the transition of inflammatory M1 macrophages to anti-inflammatory M2 macrophages (Yokota et al., 2000; Yamaguchi et al., 2005; Ohashi et al., 2010). Regulation of adiponectin on macrophages may be involved in the regulation of the islet microenvironment, thereby inhibiting β-cell inflammation. On the other hand, exercise suppresses resistin, which is an adipocytokine involved in escalating inflammation and peripheral insulin resistance (Steppan et al., 2001; Balducci et al., 2010). Although the data up-till-now is still insufficient to confirm the contribution of adiponectin and resistin on β-cell inflammation and function, it may be a viable study topic in the future.

The inhibitory effect of exercise on variables that escalate inflammatory response, such as liver-derived fetuin-A, might also reflect the protective effect of exercise on insulin secretion. Exercise can efficiently decrease circulating fetuin-A levels (Gonzalez-Gil and Elizondo-Montemayor, 2020). Fetuin-A activates TLR4, which is a key receptor that is involved in inflammation and aggravates lipotoxicity (Pal et al., 2012), resulting in impaired glucose sensing and GSIS (Shen et al., 2015) in a probable JNK and Ca^2+^-dependent manner (Gerst et al., 2017).

### Exercise enhances β-cell glucose-stimulated insulin secretion

Exercise-derived secretion factors such as IL-6, brain-derived neurotrophic factor (BDNF), and C-X3-C motif chemokine ligand 1 (CX3CL1) are likely to affect insulin secretion both directly and indirectly in a variety of pathways. IL-6 is reported to enhance GSIS potentially via a PLC–IP3-dependent pathway (Suzuki et al., 2011). In addition, Ellingsgaard et al. (2011) observed that muscle-secreted IL-6 could stimulate intestinal L cells to secrete GLP-1, which in turn acts on β-cells to promote insulin secretion. This observation is in accordance with another finding that serum GLP-1 concentrations increase significantly in both healthy and T2D subjects after exercise (Lee et al., 2015). Furthermore, considering the engagement of GLP-1 in β-cell proliferation (Muller et al., 2019), exercise may also modulate β-cell proliferation via the IL-6–GLP-1 pathway.

BDNF may also play a role in the regulation of insulin secretion during exercise. Although there is no direct evidence that skeletal muscle secretes BDNF, studies have shown that exercise can stimulate BDNF expression in skeletal muscle (Gómez-Pinilla et al., 2001). BDNF was originally discovered to act on the central nervous system and influence a variety of neurobiological processes. However, it has recently been shown that BDNF enhances the GSIS of β-cells possibly by enhancing the calcium response required for insulin secretion after acting on its β-cell-located receptor, TrkB.T1 (Fulgenzi et al., 2020).

In 2013, Lee et al. (2013) discovered that CX3CL1 promotes glucose- or GLP-1-stimulated β-cell insulin secretion in a CX3CR1 and MEK-dependent way, and they also suggested the possible role of exogenous CX3CL1 in promoting insulin secretion and maintaining β-cell function. Later, the presence of CX3CL1 stimulated by exercise in skeletal muscle was confirmed (Strömberg et al., 2016), implying that CX3CL1 may hopefully act as a mediator of exercise to improve pancreatic secretory function. Still, the pathway of skeletal muscle-derived CX3CL1 from the muscle to the bloodstream and eventually to β-cells needs to be clarified.

Moreover, leptin was identified as an adipocyte-producing peptide hormone that is required for body weight regulation and a variety of other properties (Zhang et al., 1994). Prior researches have suggested an association between exercise and leptin levels. According to systematic reviews of randomized controlled trials, exercise may decrease blood leptin levels (Balducci et al., 2010; Becic et al., 2018; Fedewa et al., 2018), suggesting that leptin could also be an exerkine. In β-cell lines, leptin has been reported to activate LEPR and in turn inhibit insulin secretion (Kieffer et al., 1996, 1997). Therefore, suppressing leptin may be another way for exercise to increase insulin secretion. However, *in vivo* data regarding such hypotheses resulted in inconclusiveness because of the relatively tiny, age- and sex-dependent role of leptin signaling in β-cell function modulation (Soedling et al., 2015). The effect of leptin on β-cells under high metabolic stress also requires further investigation.

### Exercise promotes β-cell survival and proliferation

Exerkines such as irisin, follistatin, osteocalcin, and FGF21 have been reported to possibly contribute to β-cell survival and proliferation. Exercise stimulates the muscular synthesis and secretion of irisin via the PGC1α–FNDC5 pathway, which has been shown to improve glucose homeostasis and relieve fasting insulin (Boström et al., 2012). Although initially found to act on adipocytes, irisin is later reported to promote β-cell survival, increase GSIS, and stimulate proliferation, especially when cells are stressed by SFAs (Natalicchio et al., 2017). Another study demonstrated that irisin could promote β-cell proliferation through the ERK–MAPK pathway and inhibit β-cell apoptosis through regulating apoptosis-related proteins, such as caspase (Liu et al., 2017b). Given the potential benefits of irisin, discovering its receptor on β-cell may provide a new druggable target to mimic the beneficial effect of physical exercise.

Osteocalcin is a bone-marrow-originated exerkine that affects β-cell proliferation. Osteocalcin levels are reported to increase following exercise in both non-obese, obese, and T2D subjects (Curran et al., 2020). Osteocalcin may act on β-cells through the Gprc6a receptor to enhance β-cell proliferation in a cyclin D1-dependent manner. Suppression of the osteocalcin/Gprc6a pathway results in reduced β-cell mass (Wei et al., 2014). Meanwhile, exogenous osteocalcin injections into a T2D mouse model dramatically boost β-cell mass (Ferron et al., 2012). Additionally, incubating pancreatic islets with osteocalcin upregulates genes related to insulin and β-cell proliferation (Ferron et al., 2008). These findings suggest that the osteocalcin pathway is another plausible approach by which exercise increases β-cell proliferation. Nevertheless, the processes by which exercise modulates osteocalcin need to be further investigated.

Besides its anti-inflammation property, adiponectin has been proved to inhibit β-cell apoptosis (Holland et al., 2011). Adiponectin overproduction in transgenic mice reduces caspase-8-mediated apoptosis, whereas adiponectin ablation in mice increases apoptosis *in vivo* via a sphingolipid-mediated mechanism (Holland et al., 2011). In the absence of AMP-dependent kinase, adiponectin potently activates ceramidase via interaction with its two receptors, AdipoR1 and AdipoR2, to accelerate ceramide degradation and production of its anti-apoptotic metabolite, sphingosine-1-phosphate (Holland et al., 2011). There is also evidence that the anti-apoptotic effect of adiponectin may be related to the improvement of lipid metabolism in β-cells (Ye et al., 2015). These results revealed a potentially interesting relationship between β-cell lipid metabolism and the sphingolipid pathway attributable to adiponectin, and a new possible pathway for the inhibition of β-cell apoptosis might arise.

Follistatin, another liver and muscle secretory factor whose production is greatly boosted by exercise, has also piqued researchers’ curiosity. Expressed also in mature β-cells (Mylonas et al., 2006), follistatin mainly binds to activin-A, a secretory protein of the transforming growth factor β family (Thompson et al., 2005), and thereby antagonizes the activin signaling pathway. Follistatin may potentially play an essential part in the survival of β-cells, where streptozotocin injections, resulting in massive cell destruction, are noted to eliminate follistatin in β-cells within 24 h (Ogawa et al., 1995). Loss of follistatin is likely to abnormally activate activin-A signaling and is reported to impair proliferation, differentiation, and function of β-cells (Zhang et al., 2004; Szabat et al., 2010). However, on one hand, there is limited evidence that muscle-secreted but not endogenous production of follistatin affects β-cell survival and reproduction; on the other hand, no definitive conclusion on the role of activin-A in β-cells has been reached, and some investigations have even come to contradictory results, necessitating future research into this mechanism.

It should be noted that dietary and exercise interventions share mechanistic pathways, in which β-cell survival and proliferation may be affected. For instance, the hepatic secretion of FGF21 is not only regulated by nutritional status but also has been proved to be stimulated by exercise (Staiger et al., 2017). In this sense, exercise may inhibit β-cell apoptosis by activating the ERK1/2 and AKT pathways with exercise-induced, liver-derived FGF21 (Wente et al., 2006). Adiponectin is likely to be another example with an indication of increased adiponectin levels possibly after exercise from extensive assessments of randomized controlled trials (Becic et al., 2018).

### Possible reversal effect of exercise on β-cell identity loss

Recent discoveries have shown the essential role of β-cell dedifferentiation and transdifferentiation in T2D pathogenesis. The effect of exerkines on the β-cell identity maintenance, however, has not been widely reported. As reported by Tanday et al. (2020), Apelin could be an exerkine that restores β-cell identity. Interestingly, evidence suggests that GABA, on the other hand, might act as a link between exercise and alterations in the islet cell differentiation state. GABAergic nerve cell bodies exist on both the periphery of islets and the islet mantle. Previous researches also revealed the close relationship between these GABAergic nerve cells and pancreatic α-cells (Sorenson et al., 1991). Recently, the relations between GABA and β-cell identity were further revealed. Exposing α-cells to GABA over an extended period of time or exciting the signal transduction of GABA's receptor has been found to induce the cell conversion from α- to functional β-like *in vivo* (Ben-Othman et al., 2017; Li et al., 2017). Notably, GABA treatment of transplanted human islets resulted in α-cell loss and a rise in β-like cell number, indicating that the conversion processes occur in humans as well (Ben-Othman et al., 2017). In the meantime, physical exercise has been suggested to promote the activity of intra-cephalic GABAergic nerves (Li and Spitzer, 2020). Whether exercise also induces GABA production in para-islet GABAergic nerves and how such induction affects β-cell identity, therefore, open an interesting topic for future discussion. The potential interaction between the nervous system and β-cells has now become a rising topic for the emerging islet neuroendocrinology, and it is likely to bring us novel insights into the molecular mechanisms of therapeutic exercise intervention.

## Conclusions and perspectives

During the pathogenesis of T2D, metabolic stress disrupts the normal functioning of β-cells, causing a switch from β-cell compensation to decompensation. While the physiological processes of pancreatic β-cell compensation and decompensation have been widely described at both tissue and cellular levels, the signaling network underlying the compensation and decompensation processes still remains unclear, particularly regarding the molecular trigger of the specific compensation-to-decompensation switch. Temporal multi-omics characterization of β-cells as associated with the pathogenesis of T2D would provide a piece of useful and in-depth information to clarify such processes. Recently, Gao et al. (2021) systematically characterized islet transcriptomic changes and β-cell functional changes along 24 weeks of HFD feeding and highlighted the role of inappropriate immune and inflammatory responses, which could serve as major driving forces of irreversible β-cell failure in the decompensation stage. Similar results were also obtained via the time-course islet RNA-Seq data generated in our unpublished work, in which we also revealed the potential role of covalent chromatin remodeling in driving the β-cell compensatory response. Nevertheless, with the generation of more omics data, systematic integration of spatiotemporal transcriptomic, epigenomic, and proteomic data would help map out the molecular trajectory along with the β-cell compensation-to-decompensation progression.

The regulation of β-cell functional alternations can be studied at multiple scales. The nutritional and exercise statuses of an individual exert effects on multiple organs. These in turn profoundly interact with β-cell and alter cell function. In this sense, studying the ‘intracellular’ and ‘intercellular’ factors that mediate this process are both of great importance. On the ‘intracellular’ scale, current clues related to the initial trigger of β-cell functional changes have been linked to three interconnected pathways, which are known as ER stress, oxidative stress, and inflammation. Though several of these pathways have been described in previous sections, a systematic understanding of how these metabolic stresses alter the β-cell function and a clear description of the identity-related transcriptional programs are still absent. Some other important questions also remain. How do β-cells choose between apoptosis and dedifferentiation in the face of intense metabolic pressure? Are there other cell fates (e.g. pyroptosis, necroptosis, ferroptosis, etc.) beyond these two choices? New technologies such as single cell transcriptomic/epigenomic profiling and CRISPR-based genome editing could provide new insights on such questions, and more generally, on how β-cells react upon these metabolic cues *in vivo*. However, technical barriers such as reducing the pollution and data occupancy of hormone-related genes in islet single-cell RNA sequencing (sc-RNA-Seq) analysis are still unsolved, preventing these new techniques from higher efficiency. On the ‘intercellular’ scale, the roles and mechanisms of secreted factor-mediated endocrine and paracrine signals in the regulation of islet microenvironment, β-cell identity, and function under both physiological and disease conditions have drawn great attention in the field. Meanwhile, a high level of attention in the β-cell biology field has been evoked to determine whether these ‘intercellular’ processes are reversible under dietary and exercise interventions. In addition, other interesting topics such as the identity and underlying mechanisms of the ‘checkpoint factors’ among ‘healthy’, ‘metabolically stressed’, and ‘functionally failed’ β-cells in the compensation-to-decompensation process of β-cell dynamics in T2D progression require further investigation.

Similarly, it has been demonstrated that both dietary and exercise interventions may protect β-cell function by alleviating metabolic stress. In our recent unpublished work, we revealed that dietary intervention preserved β-cell GSIS function possibly by attenuating β-cell ER stress and oxidative stress. However, how exactly physical exercise affects the stress milieu in the pancreas remains largely unknown. Also, the alterations of the immune microenvironment under both dietary and exercise interventions are yet to be defined, for instance how STING signal and how immune cell percentage or function are altered. In such a case, systematic characterization of β-cell dynamics under dietary intervention and physical exercise would provide new insights. In addition, multiple immune cell types have been identified in islets using sc-RNA-Seq, among which the interaction between some of these cells has been reported in NOD mice as a critical factor of immunological tolerance in type 1 diabetes pathogenesis (Yuan et al., 2017b). It would then be interesting to map out the interactome among different cell types in the islet microenvironment, especially the interaction between immune cells and hormone-secreting cells during T2D pathogenesis. Furthermore, whether and how these interactions are regulated by dietary intervention and physical exercise should be another interesting topic for future study. Notably, recent studies from other and our groups suggest that the cGAS–STING pathway may function as a critical link among metabolic stress signals, immune responses in the islet microenvironment, and β-cell functional dynamics during T2D disease progression and dietary/exercise interventions-induced remission. Further studies in this direction will help elucidate the network in this process.

With respect to the diverse effects of different nutrients on β-cell function, one direction of dietary intervention research is the study of β-cell ‘nutriomics’, which explores the dose- and temporal-specific effects of different nutrients on β-cells. Additionally, how and why the time and frequency of eating affect β-cell metabolic stress should also be further explored. Since the regulation of glucose homeostasis is a systemic and comprehensive process with β-cells and other metabolic tissues actively involved, future research advances in the aforementioned areas in β-cells as well as in the metabolic regulation of other major metabolic tissues will provide more mechanistic insights. Benefited by the insights, novel therapeutic strategies might be invented for the prevention and treatment of T2D and its associated metabolic complications.
